# A preliminary investigation into the design of pressure cushions and their potential applications for forearm robotic orthoses

**DOI:** 10.1186/s12938-017-0345-8

**Published:** 2017-05-08

**Authors:** N. Alavi, S. Zampierin, M. Komeili, S. Cocuzza, S. Debei, C. Menon

**Affiliations:** 1 Menrva Research Group, School of Mechatronic Systems Engineering and Engineering Science, Simon Fraser University, 250-13450-102 Avenue, Surrey, BC V3T 0A3 Canada; 2 Department of Industrial Engineering, University of Padua, Via Venezia 1, Padua, Italy; 3 CISAS-Center of Studies and Activities for Space, University of Padua, Via Gradenigo 6/a, 35131 Padua, Italy

**Keywords:** Polymer, Force, Air, Wearable, Sensors, Polydimethylsiloxane (PDMS), Rehabilitation, Exoskeleton

## Abstract

**Background:**

Load cells are often used in rehabilitation robotics to monitor human–robot interaction. While load cells are accurate and suitable for the stationary end-point robots used in rehabilitation hospitals, their cost and inability to conform to the shape of the body hinder their application in developing affordable and wearable robotic orthoses for assisting individuals in the activities of daily living. This exploratory work investigates the possibility of using an alternative technology, namely compliant polymeric air cushions, to measure interaction forces between the user and a wearable rigid structure.

**Methods:**

A polymeric air cushion was designed, analyzed using a finite element model (FEM), and tested using a bench-top characterization system. The cushions underwent repeatability testing, and signal delay testing from a step response while increasing the length of the cushion’s tubes. Subsequently, a 3D printed wrist brace prototype was integrated with six polymeric air cushions and tested in static conditions where a volunteer exerted isometric pronation/supination torque and forces in vertical and horizontal directions. The load measured by integrating data recorded by the six sensors was compared with force data measured by a high quality load cell and torque sensor.

**Results:**

The FEM and experimental data comparison was within the error bounds of the external differential pressure sensor used to monitor the pressure inside the cushion. The ratio obtained experimentally between the pressure inside the pressure cushion and the 8 N applied load deviated by only 1.28% from the FEM. A drift smaller than 1% was observed over 10 cycles. The rise times of the cushion under an 8 N step response for a 0.46, 1.03, and 2.02 m length tube was 0.45, 0.39, and 0.37 s. Tests with the wrist brace showed a moderate root mean square error (RMSE) between the force estimated by the pressure cushions and the external load cells. Specifically, the RMSE was 13 mNm, 500 mN, and 1.24 N for forearm pronation/supination torque, vertical force, and horizontal force, respectively.

**Conclusions:**

The use of compliant pressure cushions was shown to be promising for monitoring interaction forces between the forearm and a rigid brace. This work lays the foundation for the future design of an array of pressure cushions for robotic orthoses. Future research should also investigate the compatibility of these polymeric cushions for data acquisition during functional magnetic resonance imaging in shielded rooms.

## Background

Robotics are playing a more prominent role in the biomedical field [1], and various robotic devices are being used for stroke rehabilitation [2]. Many of these robotic devices include wearable orthoses as parts of exoskeleton systems [3] or as hand-held devices, such as those used for grasping, that are end effector-based [4]. These systems have been developed world-wide for rehabilitation protocols that monitor the user’s improvement using feedback sensors that measure various metrics from the user via signal processing and data acquisition software [5]. Sensors such as force sensing resistors (FSRs) [6], capacitive force sensors [7], load cells [8], and torque sensors [9] are integrated into these systems to measure the applied forces that are exerted on the mechanical system. Conventional force sensors, such as FSRs mounted on an orthosis, should not come into direct contact with the user’s arm due to their thin profile. Alternative methods for measuring arm forces through muscle activity, such as electromyography (EMG) [10], can sometimes be too sensitive to environmental conditions such as electric and magnetic noise, and have small signal to noise ratios [11].

The need for a compliant force sensor that does not have the drawbacks described above led to our investigation of using a polymer to make a pressure sensor that can also withstand the load of an arm for sensing input forces [12]. Ideally, a lightweight wrist brace would have these compliant force sensors mounted on the wrist brace’s inside surface, such that they would be in contact around the forearm. This wrist brace would allow the direct measurement of forces, provide enough flexibility to conform to the surface that is applying the forces, and be portable. These features are important considerations in designing exoskeletons for rehabilitation [13]. The usability of the cushion is also highlighted by taking into account that the cushions do not need pre-pressurization, and that the software performs an automatic calibration before beginning a test. This calibration process is done by averaging the first 20 data samples that are read by the pressure sensor and then storing this value into an initialization variable. This value is used to offset the signal and bring it to an initial value of zero. This process is repeated for each pressure sensor. These usability features are important for the potential application of cushions in developing in-home therapy devices for clinical physicians to prescribe for individuals with stroke [14]. Finally, comfort for the user is a key function of the cushion system because individuals may be wearing devices that these cushions are mounted on for long periods of time [15]. The cushions are comfortable and support individuals who train with wearable devices, facilitating their rehabilitative progress while avoiding unnecessary fatigue and stress [16]. Biocompatible polydimethylsiloxane (PDMS) air cushions are also very safe to use, and so are ethically sound for use on human participants who are wearing an orthosis to measure arm movements for stroke rehabilitation [17].

In this paper, a structural configuration of a polymeric cushion is proposed to measure the interaction forces between a human and a robotic orthosis, at the forearm. The proposed design allows for the direct measurement of applied forces and is compliant, portable, and able to withstand the load of an arm. A finite element model (FEM) is used to simulate changes in air pressure when forces are applied to the top surface of the cushion; this is done in order to verify the concept idea of the cushion as a force sensor when compared to experimental repeatability testing of the cushion. To demonstrate a practical application, six cushions are mounted on a wrist brace exoskeleton, where the cushions measure the applied forces resulting from the isometric forces of the forearm inside the wrist brace. The results of this study show a proof of concept for force sensing with polymeric cushions on exoskeletons.

## Proposed configuration and model

There are several important factors to be considered when designing a polymeric cushion: (1) the contained air should be compressible inside a flexible bladder, (2) and the bladder should support its own shape when not loaded with an external force, (3) the material of the bladder should return to its initial shape after being loaded and unloaded without tearing or plastic deformation, (4) the material of the bladder should be biocompatible with human skin, (5) the cushion should be easily and inexpensively fabricated in a lab setting, and (6) the cushion configuration should be considered for connecting the cushion to an external pressure sensor that is read by a data acquisition system and processed by the software of the robotic system.

**Fig. 1:**
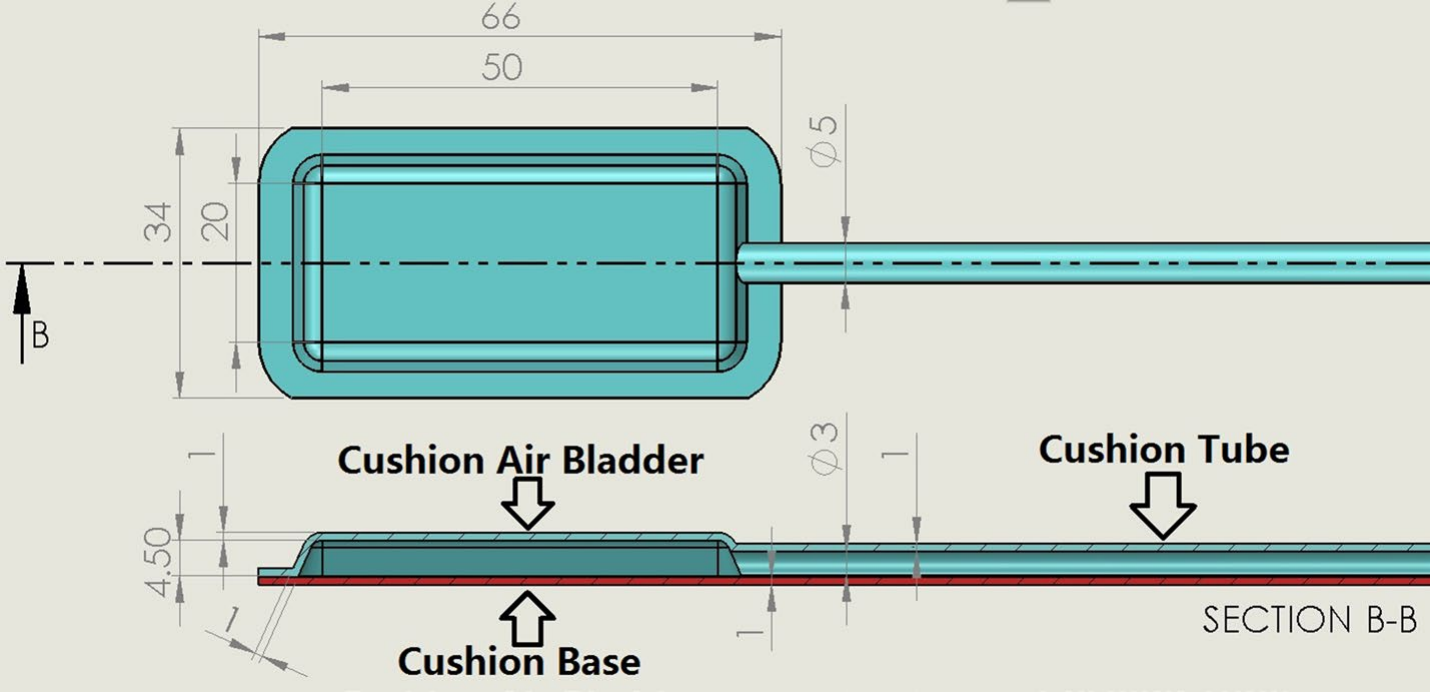
Drawing of the cushion bladder (*blue*) and the cushion base (*red*), dimensions in millimeters

The proposed cushion design is an enclosed chamber with an outlet connected to a tube that connects to an external pressure sensor. The entire cushion is designed to be made of two pieces of PDMS; one piece is a cushion air bladder (blue in Fig. 1), and the other is a cushion base (red in Fig. 1). The cushion tube, with a wall thickness of 1 mm and an air chamber of 3 mm, is drawn 200 mm out from the cushion air bladder. The 4.5 mm tall air chamber of the bladder, with a wall and ceiling thickness of 1 mm, standing on top of the 1 mm thick PDMS cushion base, results in a total cushion height of 6.5 mm.

To thoroughly understand the behavior of our proposed cushion, an FEM was created using ANSYS Mechanical APLD 14.0 [18]. The geometry of the cushion was drawn and represented by a simplified rectangular chamber, 50 × 20 × 6.5 mm^3^, with a narrow cylindrical tube, 200 mm long and 5 mm in height with an inner diameter of 3 mm, connected to the main chamber. This chamber was modeled to contain air as an ideal gas, at atmospheric pressure and at a standard room temperature of 25 °C. A flat surface was drawn to represent a solid object pressing onto the top surface of the cushion. This flat surface was modeled with material properties similar to those of an aluminum block, which is more rigid compared to the cushion material. As the surface of the aluminum plate is pressed onto the cushion, the pressure of the contained air inside of the cushion increases due to the reduction in volume of the cushion chamber. The FEM was created using a script that was written with ANSYS command line codes. The cushion was analyzed using a linear model because the deformation of the material used to prototype the polymeric cushion, the Sylgard 184 PDMS [19], was within the linear region of its stress–strain curve and elastic modulus-strain curve [20].

**Fig. 2:**
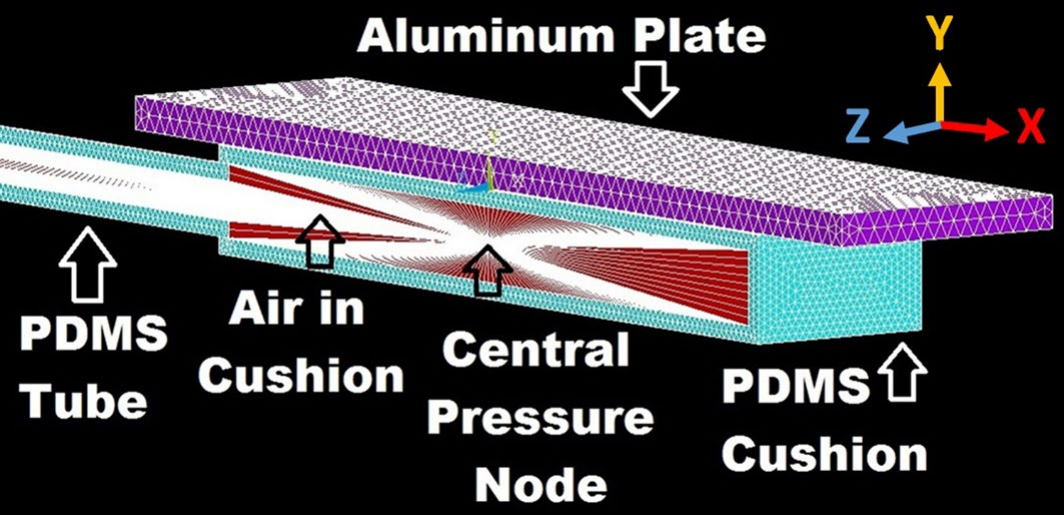
ANSYS mechanical APDL model, symmetrically cross-sectioned along the x-axis

Simulations were performed in order to interpret the experimental results which were obtained by loading the cushion with an aluminum plate. In order to simulate the air contained inside the cushion, the air was modeled such that as the fluid’s volume or temperature changed, it caused a change in the pressure exerted onto the walls of the structure. Single lines were used to connect the nodes on the inner walls of the chamber to a shared ‘central pressure node’ located at the geometric center of the cushion bladder. This allowed the change in volume of the cushion to produce a uniform pressure inside the cushion bladder. The model was drawn and meshed with its respective materials; purple elements represent the aluminum plate, the blue elements represent the PDMS cushion and tube, and the red elements represent the air inside the cushion, as seen in Fig. 2.

The white cross seen in the middle of the cushion and the white triangle seen in the middle of the cushion tube are due to the geometry of the cushion mesh having a higher concentration of air elements at these locations when connecting the inner walls of the cushion to the central pressure node in the middle of the cushion bladder. Since the line segments of the element edges are white and the elements are very close, the line segments start to appear as a uniformly white shape.

The boundary conditions that were applied onto the cushion were made to simulate the cushion’s behavior and account for the simplification of the model compared to the actual cushion. The bottom surface was fixed in place to represent the cushion being mounted on a rigid surface. The end of the air tube was fixed in place to represent being attached to the inlet of an external air pressure sensor. The top surface of the aluminum plate was given a displacement value. The aluminum plate was lowered onto the top surface of the cushion until it came into contact, and then compressed the cushion up to 0.2 mm with 50 µm step increments. As the top of the PDMS cushion was pressed by the aluminum plate, the inner volume of the cushion’s bladder was reduced, which compressed the air inside the bladder and increased the pressure from its initial atmospheric pressure. This change in pressure was measured at the central pressure node.

In order to simplify the model, the model was split in half along the xy-plane of symmetry through the aluminum plate, the cushion bladder, and the air tube as seen in Fig. 2. The symmetrical boundary plane of the model was free to move in the horizontal x-axis and vertical y-axis, but was kept fixed along the perpendicular z-axis.

## Cushion fabrication

**Fig. 3:**
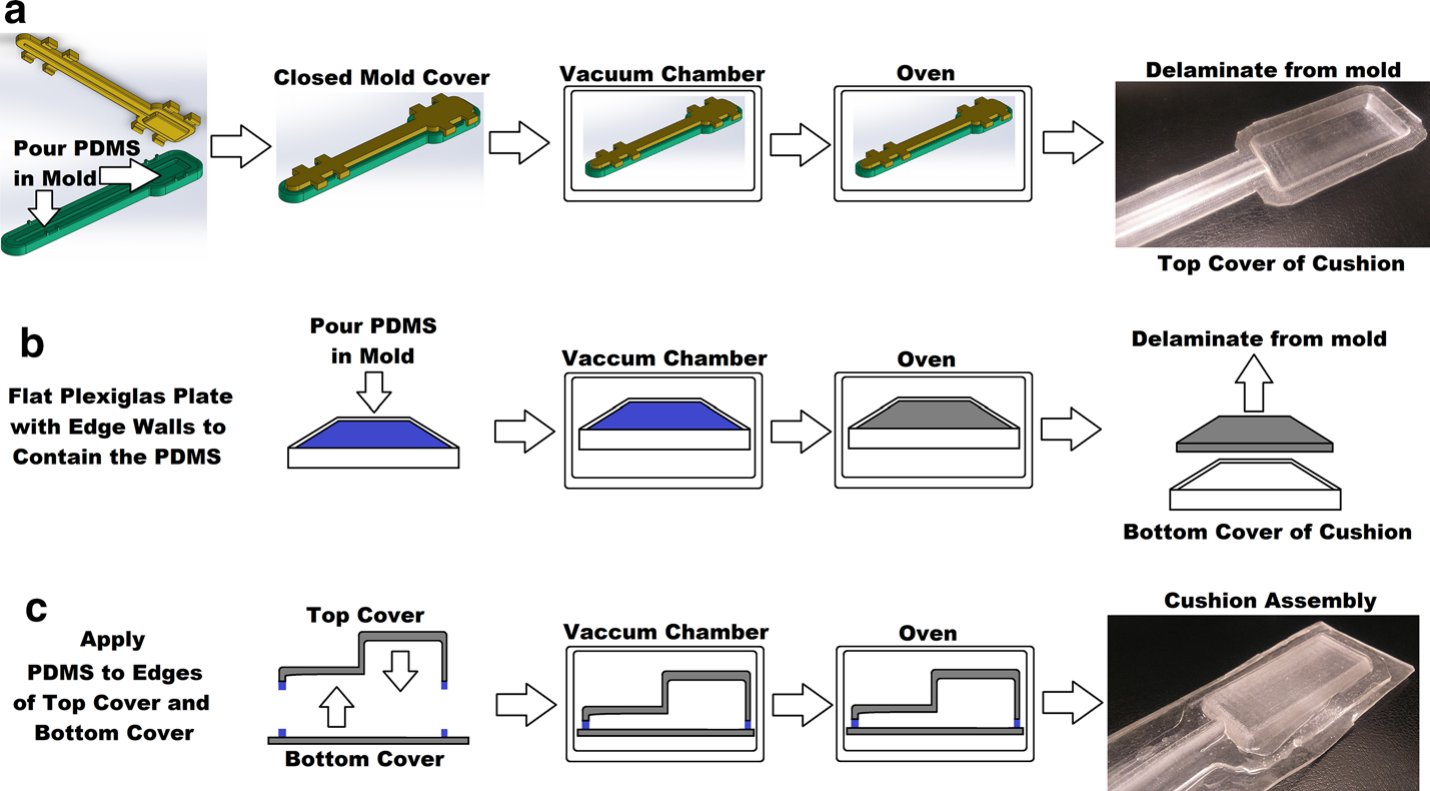
Fabrication process of the **a** top cover, **b** bottom cover, and **c** cushion assembly

Polydimethylsiloxane was chosen for the polymeric cushion due to its material properties, as well as because it is easy to fabricate from inexpensive equipment. Rapid prototyping was feasible and convenient using the available equipment in our lab [21]. The 3D model of the two-piece mold was drawn in SolidWorks and 3D printed in-house with a Fortus 250mc by Stratasys out of ABSplus plastic, using the fabrication process as shown in Fig. 3.

The PDMS was prepared at a 10:1 ratio with respect to the bulk material and cross-linking material components, and then poured into the mold. The top and bottom mold pieces were then fitted together and placed in a vacuum chamber to be degassed of air bubbles for 30 min at −80 kPa. After the mixed PDMS was degassed, the mold was placed in an oven for 3 h to cure the PDMS at 80 °C. When the curing process was complete and the cushion material was cooled to room temperature, the solidified PDMS was delaminated from its mold, and formed the top cover of the cushion. This top cover was placed onto a thin 1 mm layer of cured 10:1 PDMS that formed the bottom cover of the cushion. The top and bottom covers were assembled by applying uncured PDMS on their interfacing surfaces, and then underwent another cycle of degassing in the vacuum chamber for 30 min at −80 kPa. After the second degassing process was complete, the top and bottom covers were bonded together by curing in the oven for another 3 h at 80 °C. Finally, once cooled to room temperature, the resulting cushion was a 50 × 20 × 6.5 mm^3^ bladder cushion with atmospheric pressure air inside. A precision knife was used to trim the excess PDMS from the edges of the cushion, and cut the end of the tube outlet to later be connected to an external air pressure sensor. The trimmed cushion was then outlined and cut with a carbon dioxide (CO_2_) laser cutter (VLS3.60, Universal Laser Systems, Inc., Scottsdale, AZ) [22] for shape uniformity and to seal the top and bottom covers together. Each PDMS polymeric cushion weights 20 g.

## Comparison of experimental data and finite element model

**Fig. 4:**
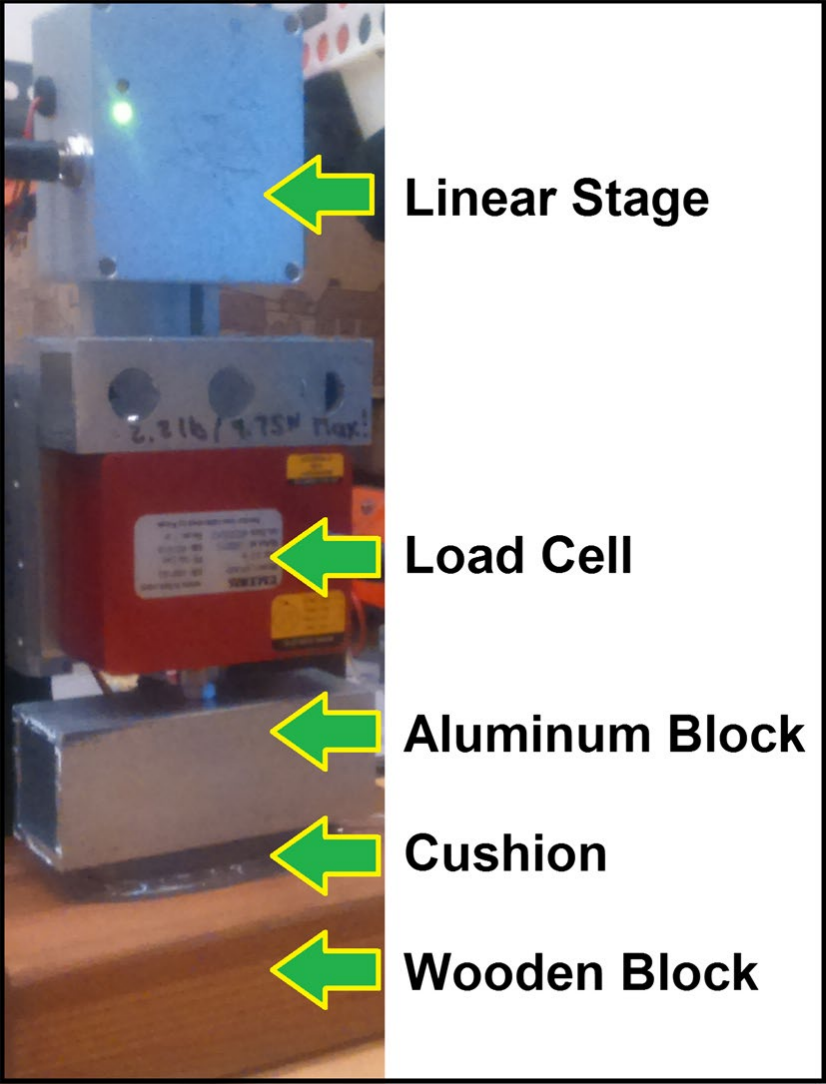
Linear stage and an aluminum block with a polymeric cushion on a wooden block

In order to accurately measure the performance of the cushion with a controllable system, a linear stage (T-LS28-SMV, Zaber Technologies Inc., Vancouver, BC) [23] was used with a load cell (LRF400 2.2 lb, FUTEK Advanced Sensor Technology, Inc., Irvine, CA, USA) [24] and a 7.2 × 3.1 × 3.1 cm^3^ aluminum block that covered the top surface of the cushion. The linear stage was controlled by a LabVIEW 2013 program that raised and lowered the aluminum block with a resolution of 100 nm, reading the force values of the load cell as the reaction force felt from the aluminum block on the cushion’s top surface, and sampling at 100 Hz. The cushion’s air tube was used to channel air from the cushion to an inexpensive $20 differential pressure sensor (MPXV7007DP, Freescale Semiconductor, Inc., Austin, TX, USA) [25] that sent the pressure signals to a data acquisition board (USB-6009 DAQ, National Instruments, Austin, TX, USA) from National Instruments [26] to be recorded by the LabVIEW program. A wooden block supported the bottom of the cushion when the linear stage pushed the aluminum block onto the top surface of the cushion, as seen in Fig. 4.

To prove the cushion’s ability to reproduce results, the PDMS cushion underwent a repeatability test. In this test, the linear stage pushed on the top surface of the cushion with the aluminum block, repeatedly loading and unloading the cushion. The tests were performed at 0.5 Hz frequency, which was considered suitable for applications regarding robotic orthoses (e.g. a recently published rehabilitation protocol based on the use of portable robotic orthoses included elbow flexion/extension cycles performed at 0.1 Hz [8]). One thousand cycles were selected, as this number greatly exceeds the number of cycles generally considered in the literature (e.g. Pignolo et al. [26] considered 200 cycles) and also greatly exceeds the expected number of cycles the cushion should consecutively be loaded in rehabilitation procedures (generally no more than a few rapid consecutive repetitions followed by a break). These 1000 cycles were repeated five times in order to evaluate if the cushions could recover their initial performance after many consecutive cycles. Repeatability tests were performed for 2, 4, 6, and 8 N loads (in total, 20,000 cycles were performed: 5 trials × 1000 cycles × 4 different loading conditions).

The LabVIEW program that was developed to perform the tests used a force-feedback system with the linear stage and load cell to accurately apply loads of 2, 4, 6, and 8 N onto the top surface of the cushion. Since the maximum limit of the load cell on the linear stage is 9.75 N (2.2 lbs) and has a nonlinearity of 0.05% of the rated output [27], therefore, 8 N was chosen as the maximum load to apply on the test cushion for the repeatability test.

**Fig. 5:**
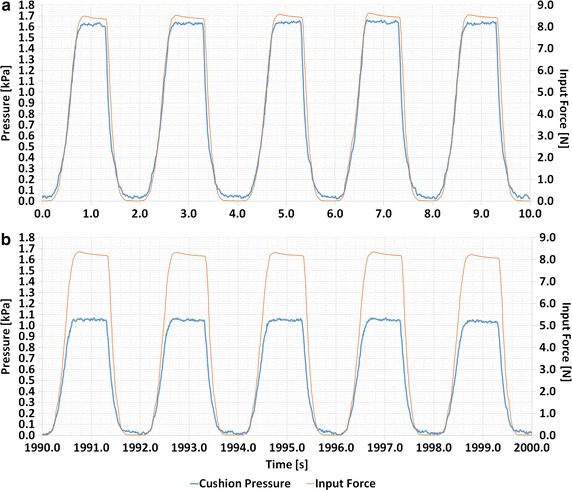
Pressure from 8 N loading of the cushion for the **a** first and **b** last 5 cycles of 1000 cycles

**Fig. 6:**
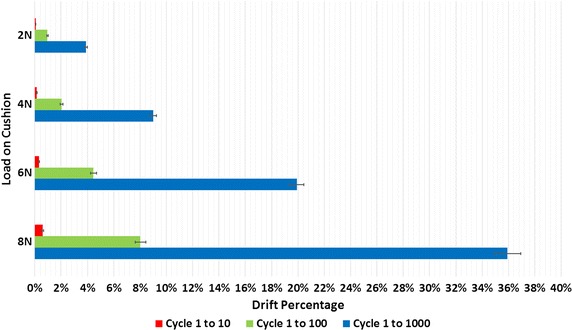
Drift percentage from the cushion load for cycle 1–10, 1–100, and 1–1000

Figure 5 shows how the pressure of the test cushion and the force from the linear stage for the 8 N load changed with time during the first 5 cycles and the last 5 cycles of a 1000 cycles test. While Fig. 5a, b qualitatively show that a large drift was present between the initial and the last cycles of a 1000 cycle test, a negligible drift was present within a five consecutive cycle range, either in the beginning or at the end of the 1000 cycle test. It should be noted that Fig. 5a, b shows the worst-case scenario, i.e. when the maximum load (8 N) was applied. Quantitative results of the repeatability test are summarized in Fig. 6. The drift percentage of the pressure is presented for the 1000 cycles (seen as the blue bars in Fig. 6) and for smaller subsets of cycles, namely for the first 10 (seen as the red bars) and 100 (seen as the green bars) cycles. The average of the drift percentage was calculated over the five 1000 trials, and was repeated under 2, 4, 6, and 8 N loads. The error bars represent the standard deviation computed out of the five different trials.

**Fig. 7:**
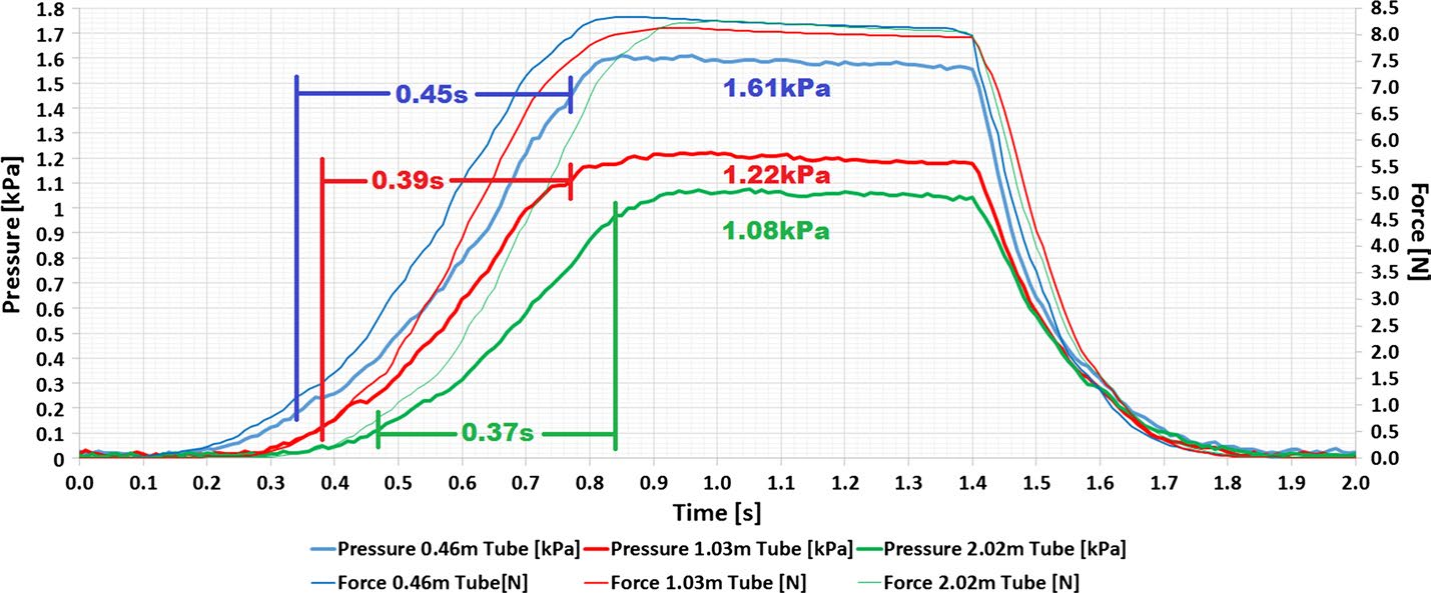
Step response of 8 N load on the cushion with short and long extension tubes

The cushion was also tested for its time delay effects with respect to an increase in the length of the cushion’s tube. For each of the time delay tests, the cushion was loaded and unloaded with a step response over a period of 2 s under an 8 N load, with the same linear stage setup as seen in Fig. 4; the tests varied only in that the length of the cushion’s tube was changed. Three extension tubes were individually attached onto the pre-existing tube of the cushion in order to test the time delay effect on the measured output pressure of the cushion with respect to each extension tube. The total cushion tube lengths were 0.46, 1.03, and 2.02 m. The step response data from the cushion is seen in Fig. 7, where the load was released at the 1.4 s mark for each test.

**Fig. 8:**
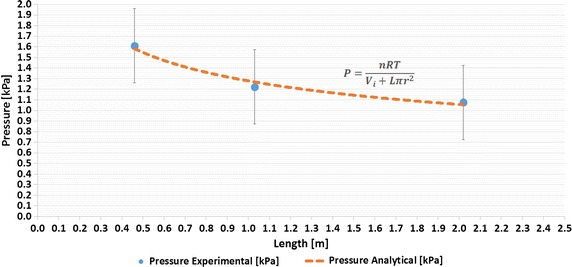
Tube length vs. cushion output pressure under an 8 N load

The peak values for the 0.46, 1.03, and 2.02 m different tube length cushion tests were 1.61, 1.22, and 1.08 kPa, respectively. The rise time, which is the time for the signal to rise from 10 to 90% of its final value [28], of each cushion’s pressure output was calculated in order for them to be compared to one another. The rise time of the 0.46 m tube was the longest at 0.45 s, the 1.03 m tube had the next shortest at 0.39 s, and the shortest rise time was from the 2.02 m tube at 0.37 s. It should be noted that the pressure of the cushion decreased by 0.39 kPa, comparing the 0.46 m tube and the 1.03 m tube, and further decreased by 0.14 kPa comparing the 1.03 m tube and the 2.02 m tube. This is due to the increase in the volume of the air in the cushion’s closed system when the length of the tube is increased. This behavior can be explained by the ideal gas law [29].

1 $$P = \frac{nRT}{V} = \frac{nRT}{{V_{i} + L\pi r^{2} }}$$

where *P* is the output pressure of the cushion, *n* is the number of particles, *R* is the gas constant, *T* is the temperature, *V*_*i*_ is the volume of the cushion bladder without the tube, *L* is the length of the tube, and *r* is the inner radius of the tube. The variables that change when the length of the tube attached to the cushion increases are the length of the tube *L* and the number of air particles *n* in the closed system. When the length of the tube is significantly increased such that *n* and *L* become very large, the *V*_*i*_ term becomes negligible, and therefore the output pressure of the cushion remains relatively the same, showing a decrease in the sensitivity of the cushion as the length of the tube increases. This behavior can been seen in Fig. 8 when comparing the length of the tube and the output pressure of the cushion. The simplified analytical model of this behavior was created using the ideal gas law from Eq. (1) and was used to characterize the correlation between the lengths of the cushion tubes used in the experimental tests and the output pressure from the cushion from an 8 N load. The resulting output pressures of the analytical model of the experimental data were plotted (see Fig. 8).

The experimental and analytical data points matched very closely and were within the ±0.35 kPa error bars of the cushion’s pressure sensor.

The data from the repeatability test was used to characterize and find a relationship between the polymeric cushion input force and its corresponding output pressure as discussed in the following paragraphs. For consistency, the experimental testing for comparing the fabricated cushion with the FEM was done using the same test cushion.

**Fig. 9:**
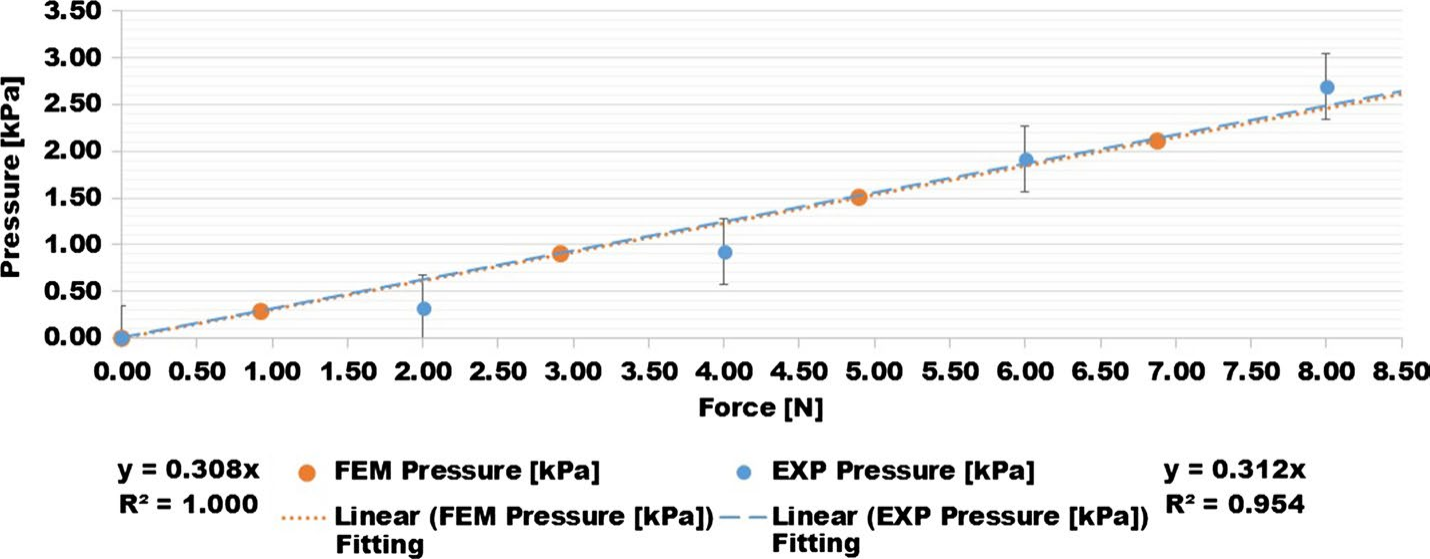
Finite element model (FEM) and experimental (EXP) force-pressure result comparison

The data points from the FEM pressure curve formed a linear relationship between the reaction force and the output pressure of the cushion as the aluminum plate pushed onto the cushion. Since the use of symmetry in the FEM gave output values that were from half of the cushion, the measured force from the FEM was multiplied by 2, and the measured pressure from the FEM was divided by 2. This allowed the FEM to be comparable with the experimental results. The structural forces were measured in ANSYS from the contact elements between the aluminum plate and the top surface of the cushion. The changes in air pressure were measured from the pressure node on the inside of the cushion. The experimental results of the aluminum block test, compared with the FEM force and pressure results, are within the ±0.35 kPa error bounds of the differential pressure sensor used to measure the experimental pressure inside the cushion, as seen in Fig. 9.

The data points of the applied forces and the air pressure for the FEM and experimental results were each fitted with a linear trend line.

2 $$P = K \cdot F$$

where *K* is the coefficient that relates the applied force on the cushion, *F*, to the measured output air pressure, *P*, from the cushion. The *K* coefficients for the experimental and FEM, as well as the linearity of regression R^2^ values of the trend lines for the polymeric cushion, are shown in Table 1.

**Table 1:** Experimental results and FEM linear trend line comparison

	K coefficient	R^2^ value
Experimental	0.312	0.954
FEM	0.308	1.000

**Fig. 10:**
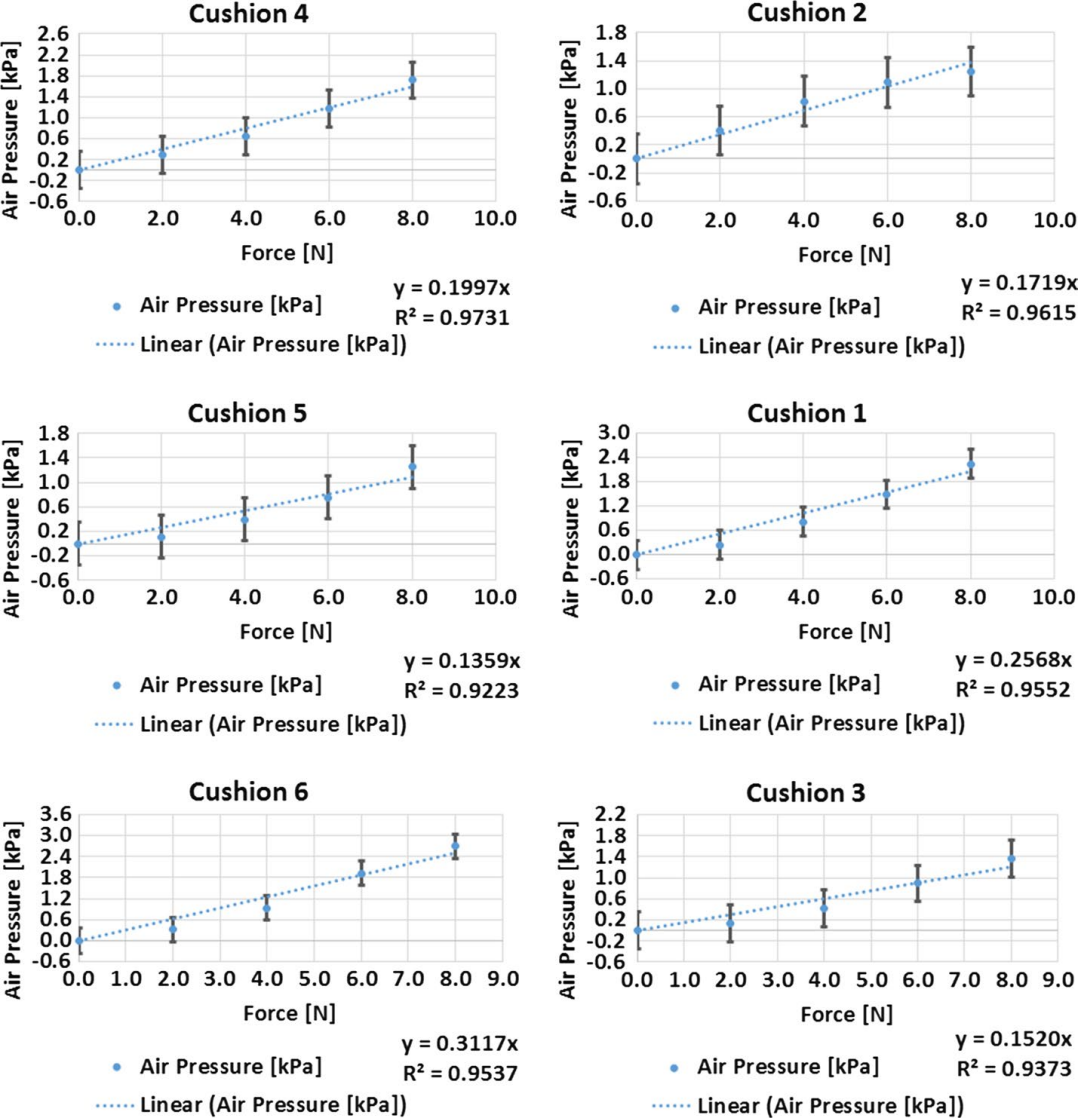
Characterization plots of force and pressure for six cushions

The R^2^ value of the experimental results and FEM trend lines are 0.954 and 1.000, respectively. The *K* coefficients representing the slopes of the trend lines between the experimental and FEM data points for the prototyped polymeric cushion have a difference of 1.28%. The formula of Eq. (2) allows the LabVIEW code to interpret the air pressure values of the cushion as an applied force. The experimental aluminum block test was repeated for five other cushions, and their respective pressure–force equations are shown in Fig. 10.

A combination of the pressure–force equations is used to measure specific isometric forces and torques at the forearm from an exoskeleton wrist brace.

## Wrist brace application

**Fig. 11:**
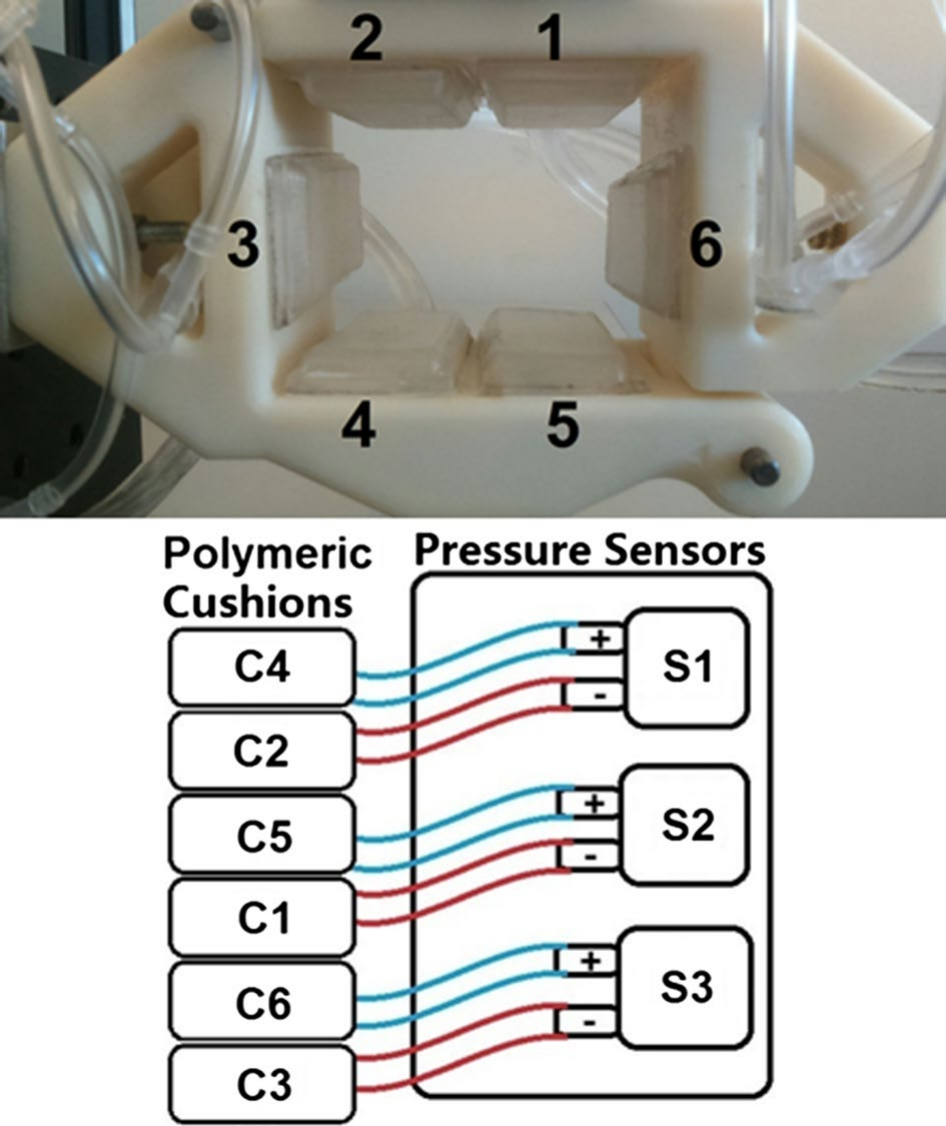
Wrist brace exoskeleton cushions labelled C1–C6, and pressure sensors labelled S1–S3

The polymeric cushions were arranged in a hexagonal configuration on the exoskeleton wrist brace in order to measure the applied forces from the forearm on the assembled exoskeleton, as shown in Fig. 11. The wrist brace was a 3D printed 9 × 7 × 7 cm^3^ structure that opened up from a hinge in order to allow the user to place their arm inside. When the latch was closed, the arm was in contact with all of the cushions, which then measured the movements of the forearm. The applied force from the user’s forearm was the input to the system, and the change in air pressure inside the cushion was the measured output. The change in pressure was measured by the differential pressure sensor read by the data acquisition card and processed by a LabVIEW program.

The air pressure sensors worked in differential pairs, as seen in Fig. 11, where the blue tube with the ‘+’ and red tube with the ‘−’ represent the positive and negative inlets of the differential pressure sensor. There are three pressure sensors, labelled S1, S2, and S3, respectively. Each pressure sensor is connected to two polymeric cushions. Thus, the S1 sensor that was attached to the polymeric cushions are labelled C4 and C2, respectively, the S2 sensor that was attached to the polymeric cushions are labelled C5 and C1, respectively, and the S3 sensor that was attached to the polymeric cushions are labelled C6 and C3. The output signals from each of the pressure sensors are the mathematical difference between each of the cushions in the pairs, where a differential change of pressure from the polymeric cushions influences the output signal of the pressure sensor to which they are attached, thus changing the output signal in the positive or negative direction. If the pressure values of the cushion pairs are equal in value, then the output signal of the pressure sensor will be zero.

Characterization tests as presented in the previous section were performed using the polymeric test cushion, which was then used as cushion C6, as seen in the cushion and sensor configuration in Fig. 11. Similar characterization curves were obtained for other cushions as well.

**Fig. 12:**
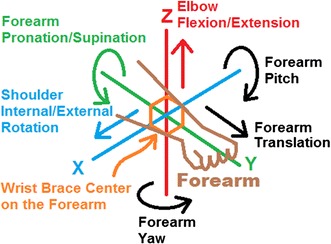
Degrees of freedom measureable by the wrist brace exoskeleton cushions

The polymeric cushions were mounted on the wrist brace to measure the user’s isometric forces for three degrees of freedom at the center of the wrist brace; in torsional forearm pronation/supination, vertical elbow flexion/extension, and horizontal shoulder internal/external rotation, as illustrated in Fig. 12 and labelled in green, red, and blue, respectively. The remaining three degrees of freedom, as labelled in black, were not measureable with the proposed cushion configuration. Forearm yaw and forearm pitch rotations would require a second row of cushions to measure the differential pressure about the x- and z-axis, respectively. Similarly, the forearm translation along the y-axis would require a cushion at the end of the user’s hand. These considerations are not included in the current design. This preliminary study of these polymeric cushions as tested on a wrist brace exoskeleton focuses on evaluating the performance of the cushions and the human interaction forces in a simple configuration to reduce the measured errors from the system. The single row of cushions around the inside of the wrist brace captures the forces applied in the xz-plane, i.e. one rotational torque and two perpendicular translation forces, as shown in Fig. 12.

Each of the possible movements can be recognized from specific combinations of force values read by the cushions on the wrist brace exoskeleton. A test setup was made to verify the accuracy of these combinations of forces from the cushions on the wrist brace exoskeleton with a load cell (LCM300, FUTEK Advanced Sensor Technology, Inc., Irvine, CA, USA) [27] and torque sensor (TRT-100, Transducer Techniques, LLC. Temecula, CA, USA) [30] while being mounted on a fixed platform. The difference between the measured cushion values on the wrist brace exoskeleton and the load cell or torque sensor reading was calculated using the following equation:

3 $$\Delta F = \left| {F_{LC} - F_{WB} } \right|$$

where *F*_*LC*_ is the force measured from the load cell, *F*_*WB*_ is the force measured from the cushions on the wrist brace exoskeleton, and ∆*F* is the absolute difference in force between the load cell and the combined forces measured from the cushions.

**Fig. 13:**
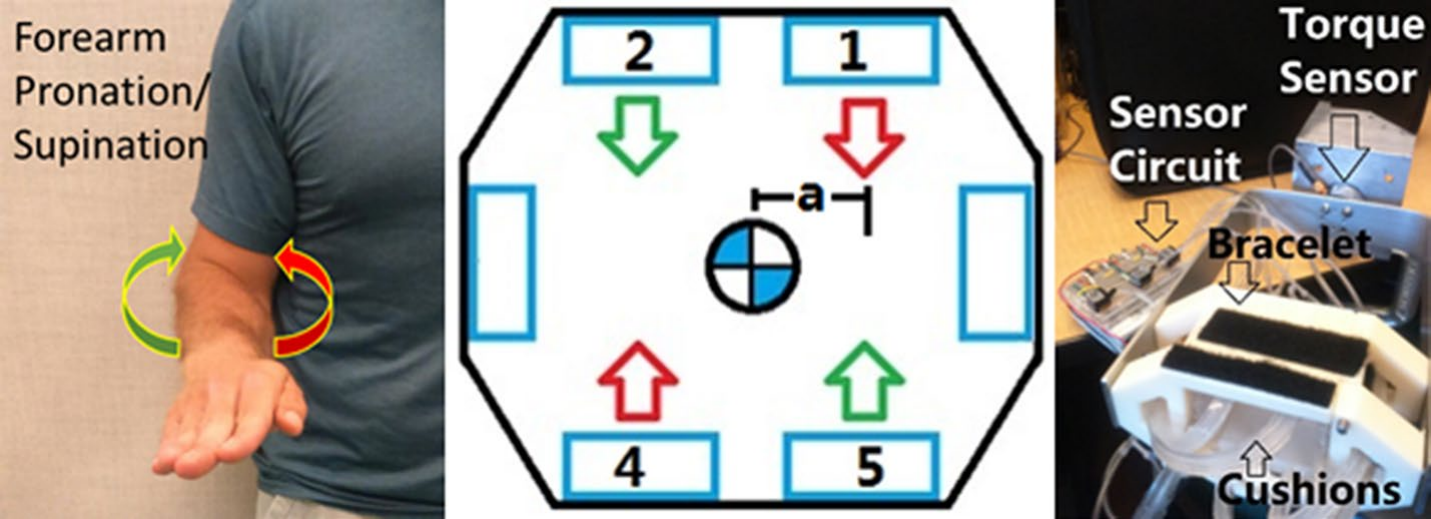
Forearm pronation/supination, wrist brace connected to torque sensor

The torsional forces applied on the wrist brace cushions from forearm pronation/supination were measured by a torque sensor. The moment arm (a) is set to 15 mm which is the distance from the axis of rotation to the force direction of the cushion surface, as seen in Fig. 13. The number 2 and 5 (green) and 1 and 4 (red) cushions were alternately relaxed and compressed, respectively, as illustrated in Fig. 13.

The torsional forces on the exoskeleton wrist brace due to the pronation/supination of the forearm were measured as follows.

4 $$T_{PS} = \left[ {\left[ {F_{C4} + F_{C1} } \right] - \left[ {F_{C5} + F_{C2} } \right]} \right]*\left( a \right)$$

where, *T*_*PS*_ is the torque of the pronation/supination, *F*_*C*1_, *F*_*C*2_, *F*_*C*4_, and *F*_*C*5_ are the forces measured from cushion 1, 2, 4, and 5, respectively, and (a) is the distance from the center of the wrist brace to the point of the applied force on the cushion surface, which is 15 mm and labelled as ‘a’ in Fig. 13.

**Fig. 14:**
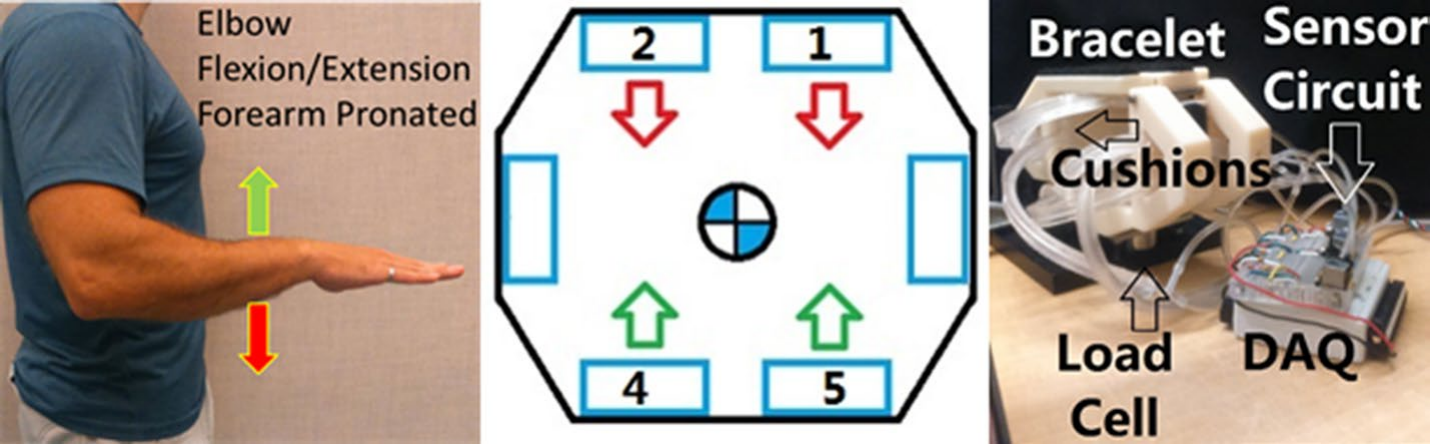
Elbow flexion/extension, forearm pronated, wrist brace connected to load cell

The vertical forces at the forearm applied on the wrist brace cushions from elbow flexion/extension were measured by a load cell, with the number 4 and 5 (green) and 2 and 1 (red) cushions being alternately relaxed and compressed as illustrated in Fig. 14.

The vertical forces on the exoskeleton wrist brace due to the flexion/extension of the elbow were measured using the following equation:

5 $$F_{FE} = \left[ {\left[ {F_{C4} + F_{C5} } \right] - \left[ {F_{C2} + F_{C1} } \right]} \right]$$

where, *F*_*FE*_ is the flexion/extension force with the elbow in the pronated position, *F*_*C*1_, *F*_*C*2_, *F*_*C*4_, and *F*_*C*5_ are the forces measured from cushion 1, 2, 4, and 5, respectively.

**Fig. 15:**
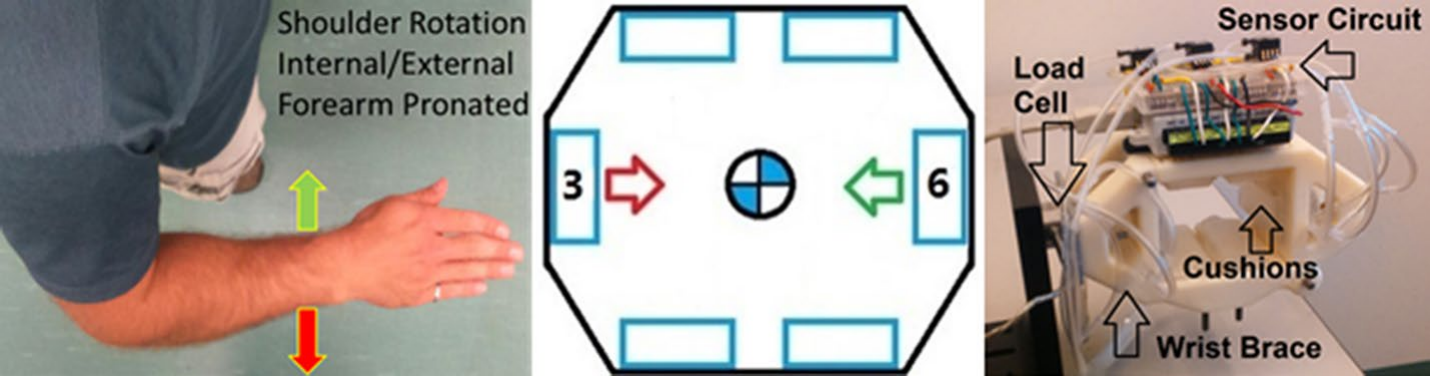
Shoulder rotation internal/external, forearm pronated, wrist brace connected to load cell

The horizontal forces at the forearm applied on the wrist brace cushions from internal/external shoulder rotation were measured by a load cell where the number 3 (green) and 6 (red) cushions were alternately relaxed and compressed as illustrated in Fig. 15.

The horizontal forces on the exoskeleton wrist brace due to the internal/external rotation of the shoulder were measured.

6 $$F_{IE} = \left[ {F_{C6} - F_{C3} } \right]$$

where *F*_*IE*_ is the force measured at the forearm from the internal/external rotation of the shoulder, *F*_*C*6_, and *F*_*C*3_, are the forces measured from cushion 6 and 3, respectively.

**Fig. 16:**
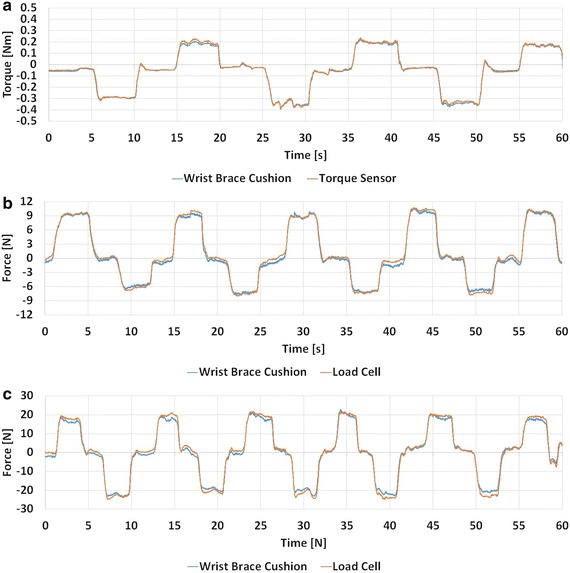
**a** Forearm pronation/supination, **b** elbow flexion/extension, **c** shoulder rotation internal/external

The results of the three forearm movements were found through processing the output signals of the differential pressure sensors and the load cell or torque sensor data through LabVIEW. The data was collected for a session of 60 s for each movement. Using the pressure–force relationships for each of the six cushions, the root mean square error (RMSE) between the measured cushion forces and the load cell or torque sensor was calculated. The plot of the torque sensor and the output from the cushions on the wrist brace exoskeleton during forearm pronation/supination is shown in Fig. 16. The red line is the combined output torque from the cushions on the wrist brace and the blue line is the data from the torque sensor.

The RMSE between the torque sensor values and the combined torque values from the cushions for pronation/supination measured at the forearm was 13 mNm. The RMSE between the load cell values and the combined force values from the wrist brace cushions for flexion/extension of the elbow and for shoulder rotation internal/external measured at the forearm was 500 mN vertically and 1.24 N horizontally.

## Discussion

Six polymeric cushions were used to measure interaction forces between a human forearm and a wrist brace. This was done using differential pressure sensors to measure changes in the air pressure inside the polymeric cushions; these changes were in turn converted into force and torque values. The maximum difference between the forces and torques measured from the wrist brace polymeric cushions and the load cell and torque sensor was 1.24 N and 13 mNm, respectively. Typical isometric strength capabilities of human joint torques have been considered in the design of many powered exoskeletons for stroke rehabilitation [31], where elbow flexion/extension, forearm pronation/supination, and shoulder internal/external rotation are reported to be 72.5, 9.1, and 6 Nm, respectively [32]. The error of the polymeric cushions is small enough to be used with exoskeletons. An example of such an exoskeleton is the Haptic Knob, a robotic end-effector device in which the user grabs the knob interface with their fingers and performs forearm pronation/supination rehabilitation exercises. The 13 mNm error from the polymeric cushions when measuring forearm pronation/supination is less than 1% of the Haptic Knob’s maximum forearm pronation/supination torque of 1.5 Nm [31]. Another example is the bimanual wearable robotic device (BWRD) system, which is a force feedback bimanual wearable elbow rehabilitation device for stroke [8]. The BWRD system’s robotic arms are able to provide a maximum theoretical torque of 18.2 Nm for the Slave arm and 13.9 Nm for the Master arm, at the elbow for flexion/extension exercises that form parts of a training protocol. Within the series of tasks defined in the protocol, task #5 requires stroke individuals to actively move both of their elbow joints together. If the force sensors detect that the difference of applied forces between the two arms is greater than 1 Nm, the BWRD applies resistance to the motion of both arms through its motor and brake system, prompting the user to correct the imbalance of the forces that they are applying on the exoskeleton arms. When the difference of applied forces is less than 1 Nm, the arms are free to move [8]. The polymeric cushions are suitable for such a task because the error in the measured torque at the elbow for flexion/extension would be approximately 144 mNm, considering that the average forearm length is 26.2 cm [33] and that the maximum error from the polymeric cushions is 500 mNm for flexion/extension of the elbow. Herrnstadt et al. [8] observed that a few participants found this task to be difficult and suggested increasing the 1 Nm threshold for some participants. Yet even at this setting, the polymeric cushions would be appropriate for measuring elbow flexion/extension forces. The robotic device made by Tsagarakis et al. for upper extremity physiotherapy and training was able to produce 6 Nm of torque for the shoulder internal/external rotation joint [34]. The RMSE of the force of the wrist brace cushions, measured at the forearm for internal and external shoulder rotation, was found to be 1.24 N. Assuming that the forearm length is 26.2 cm, the torque error would be 0.32 Nm, which is about 5% of the torque value reported for the device by Tsagarakis et al. [32]. The configuration and design of the cushions, which are made completely from PDMS and air, also allow for new opportunities of measuring interaction forces between a human and an orthosis, such as in magnetoencephalography (MEG) rooms [35] where conductive materials are prohibited while measuring brain activity using highly sensitive electromagnetic waves. Mounting these cushions onto a metal-free pneumatic exoskeleton allows for stroke assessment in an MEG room, provided that the air pressure sensors connected to the cushions’ tubes are placed in the control room.

Although a significant drift of up to 36% for the 8 N load was observed after 1000 cycles, typical real-world applications would experience far less signal drift because 1000 cycles is beyond the number of repetitions that are conducted within a single bout of repetitions within a rehabilitation session. The drift after 10 consecutive rapid cycles has a more realistic significance—exercise sessions with periods of 10 cycle repetitions followed by breaks are, in fact, reflective of real-world applications, such as the one that Masiero et al. [36] performed. The signal drift after 10 consecutive cycles was found to be always less than 1%. Negligible variation between the five different trials was observed (standard deviation: <0.05%) as seen by the error bar on the 8 N load for cycle 1–10.

The effects of the length of the cushion’s tube on the response of the cushion’s output pressure have been investigated and characterized for three tube lengths, 0.46, 1.03, and 2.02 m, with a step response under an 8 N load. The rise time was always less than 0.5 s for all tube lengths, showing that the pressure sensors could potentially be used in shielded rooms. Our results show also that as the length of the tube increases, the resulting output pressure from the cushion decreases due to the increase in the volume of air in the cushion’s closed system. As shown in Fig. 8, it can be seen that as the tube’s length increases, the output pressure decreases. This phenomenon can be predicted using a simple analytical model based on the ideal gas law.

## Conclusions

In this paper, the use of air pressure polymeric cushions to measure interaction forces between a human and a wrist brace exoskeleton was studied. The polymeric cushions were made from PDMS and were connected to differential air pressure sensors. The completely non-conductive cushions could be connected to a very sensitive and high quality pressure sensor of the user’s choosing. Six polymeric cushions were mounted onto the interface surface of a wrist brace exoskeleton and were to be in direct contact with the user’s arm to measure the interaction forces; they also were light weight, functionally easy to use, and could comfortably conform to the shape of a user’s arm. The rectangular top surface of the polymeric cushions, which came into contact with the forearm, was made such that one polymeric cushion could fit along the side of the forearm. The polymeric cushions were fully in contact with the user’s forearm, and allowed for direct measurement of the forces applied from the forearm onto the wrist brace exoskeleton, thus reducing the force measurement error. The 20 g weight of an individual polymeric cushion allows it to be portable and versatile.

The test cushion ran through a repeatability test, during which 5 trials of loading/unloading cycles were repeated for 2, 4, 6, and 8 N loads. For 10 consecutive rapid (0.5 Hz) cycles, the drift percentage from the cushion was less than 1% in the worst case scenario that was considered (8 N load). By comparing the five trials, a small standard deviation (i.e. <0.05%) for the drift was recorded for 10 cycles.

A tube length test was performed to test the time delay effects of a longer tube. The rise time was smaller than 0.5 s for tubes between 0.5 and 2 m. The relationship between the tube length and the output pressure of the cushion was experimentally measured and compared to an analytical model using the ideal gas law. The experimental data and analytical model were closely related and within the error bars of the pressure sensor. An FEM of the cushion was made to simulate its compression from a load onto its top surface. This was similar to when the cushion was loaded with an arm when the wrist brace exoskeleton was worn. A test cushion was made and subjected to the same loads as simulated in the FEM. The comparison between the FEM and experimental results showed the difference to be within the error of the differential pressure sensors used in the experimental results. The wrist brace exoskeleton was equipped with six individual cushions and configured to measure the isometric forces of the torque due to forearm pronation/supination, as well as isometric vertical forces due to the flexion/extension of the elbow, and isometric horizontal forces due to the internal/external rotation of the shoulder. A test was performed to compare the forces and torques measured by the wrist brace exoskeleton cushions with those of an off-the-shelf torque sensor and load cell. When comparing our cushions to the off-the-shelf load cell and torque sensor, the RMSE of the isometric torques and forces was found to be 13 mNm for forearm pronation/supination, 500 mN for vertical forces, and 1.24 N for the horizontal forces.

Future work involves the exploration of different shapes and sizes of cushions that may be used to measure movements of other parts of the body, as well as optimizing the cushions’ dimensions to maximize the sensitivity of the cushions. The configuration of the polymeric cushions on the wrist brace exoskeleton could have additional sensors to fully measure the interaction forces in all 6° of freedom. Other future work may involve more intensive testing with the cushions being worn for longer periods of time, testing for hysteresis, and performing end-of-life tests for the polymeric cushions.

## Abbreviations

BWRD: bimanual wearable robotic device
EMG: electromyography
FEM: finite element model
FSR: force sensing resistor
MEG: magnetoencephalography
PDMS: polydimethylsiloxane
RMSE: root mean square error
